# A Low-Noise Transimpedance Amplifier for BLM-Based Ion Channel Recording

**DOI:** 10.3390/s16050709

**Published:** 2016-05-19

**Authors:** Marco Crescentini, Marco Bennati, Shimul Chandra Saha, Josip Ivica, Maurits de Planque, Hywel Morgan, Marco Tartagni

**Affiliations:** 1 Department of Electrical Electronic and Information Engineering G. Marconi, University of Bologna, Cesena Campus, Via Venezia 52, IT-47521 Cesena, Italy; marco.tartagni@unibo.it; 2 Advanced Research Center on Electronic Systems (ARCES), University of Bologna, Cesena Campus, Via Venezia 52, IT-47521 Cesena, Italy; 3 Center for Industrial Research (CIRI-ICT), University of Bologna, Via Venezia 52, IT-47521 Cesena, Italy; marco.bennati@unibo.it; 4 Department of Electronic and Computer Science & Institute for Life Science, University of Southampton, University Road, SO17 1BJ Southampton, UK; shimul.saha@mediwise.co.uk (S.C.S.); josip.ivica87@gmail.com (J.I.); mdp@ecs.soton.ac.uk (M.d.P.); hm@ecs.soton.ac.uk (H.M.)

**Keywords:** transimpedance amplifier, current sensing circuit, low-noise amplifier, low-noise current sensor, noise, ion channel recording, bilayer lipid membranes, electrophysiology

## Abstract

High-throughput screening (HTS) using ion channel recording is a powerful drug discovery technique in pharmacology. Ion channel recording with planar bilayer lipid membranes (BLM) is scalable and has very high sensitivity. A HTS system based on BLM ion channel recording faces three main challenges: (i) design of scalable microfluidic devices; (ii) design of compact ultra-low-noise transimpedance amplifiers able to detect currents in the pA range with bandwidth >10 kHz; (iii) design of compact, robust and scalable systems that integrate these two elements. This paper presents a low-noise transimpedance amplifier with integrated A/D conversion realized in CMOS 0.35 μm technology. The CMOS amplifier acquires currents in the range ±200 pA and ±20 nA, with 100 kHz bandwidth while dissipating 41 mW. An integrated digital offset compensation loop balances any voltage offsets from Ag/AgCl electrodes. The measured open-input input-referred noise current is as low as 4 fA/√Hz at ±200 pA range. The current amplifier is embedded in an integrated platform, together with a microfluidic device, for current recording from ion channels. Gramicidin-A, α-haemolysin and KcsA potassium channels have been used to prove both the platform and the current-to-digital converter.

## 1. Introduction

**Figure 1:**
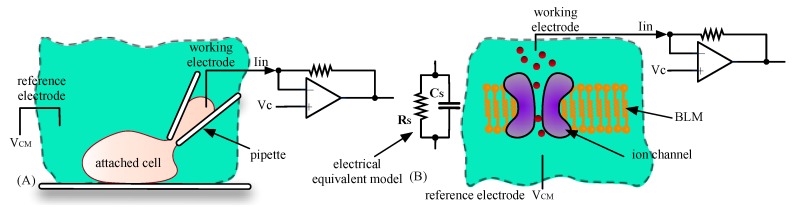
(**A**) Diagram showing the patch clamp technique where a glass pipette is used to pull a patch of cell membrane. A low-noise transimpedance amplifier measures channel currents; (**B**) the planar bilayer membrane (BLM) technique where a suspended lipid bilayer contains an ion-channel. Again the current is read by a low-noise transimpedance amplifier. The picture also shows the electrical equivalent model of the BLM, consisting of a high value resistor (of the order of GΩ or greater) in parallel with a capacitance *C_S_*.

Ion channels are nanoscale pores that sit in the cell membrane, allowing communication of the cell with the external environment through ionic currents. The open/close behavior of ion channel is modulated through different mechanisms, e.g., voltage, ligand binding, pH change, or mechanical strain. Channels are crucial for the control of physiology and any malfunction is at the root of a variety of pathologies and diseases [1]. Ion channel recording is an important component of the next generation HTS diagnostic tools used for drug discovery, DNA sequencing and single molecule detection [2]. There are two main techniques for ion channel screening:

- Patch clamp, where a glass pipette, or a micro-aperture in a solid-state device is used to pull a patch of cell membrane [1,3,4] (Figure 1).
- Planar bilayer lipid membranes (BLM), where a single ion channel is inserted into a lipid bilayer suspended over a micro-aperture [5] (Figure 1).

The patch-clamp technique is widely used in modern HTS instruments. The advantages of patch-clamp are high fidelity, since the ion channels exist in their native physiological environment, together with a high level of automation and parallelization [4,6]. This technique suffers from low specificity and high noise since a number of different ion channels are measured together, and also the membrane provides a large capacitance. On the contrary, BLM technique provide excellent electrical sealing and high sensitivity detection, down to single molecule, with minimum noise and capacitance [7,8,9]. The design of HTS system based on BLM ion channel recording faces three main challenges [10]:

- A microfluidic device allowing stable, reliable and automatic BLM formation.
- A fast low-noise electronic interface ables to acquire pA currents.
- A compact, robust and scalable system containing an array of microfluidic devices and electronic interface.

This paper focuses on the second challenge above; the other two challenges have been discussed previously [11,12,13]. The electronic readout is a key element in the design of a BLM-based HTS system. The main requirements for the electronic interface are low-noise (noise floor <10 fA/√Hz), high-sensitivity (transresistance >1 GΩ) and wide-bandwidth (>10 kHz) [14,15]. Specific requirements are mainly related to the kind of ion channel under investigation. For instance, potassium ion channels, such as KcsA, have fast responses (100 μs) and zero-voltage conductivity lower than 100 pS, resulting in currents of the order of a few pA with applied voltages lower than 100 mV [16]. In general an ion channel has (i) very high output impedance (from 1 to 100 GΩ); (ii) noise level smaller than 1 pArms at 1 kHz; (iii) open/close events ranging from few milli-seconds to hundreds of micro-seconds and (iv) capacitance of the order of tens of pF [10,15,17].

The benchmark for low-noise low-current recording is the Axon Axopatch 200B, which has 100 kHz bandwidth and input-referred noise of 6 fA/√Hz in resistive mode and of 0.7 fA/√Hz in capacitive mode, but it is a bulky instrument not suitable for parallel recording [18]. A great number of low-noise low-current readout circuits have been presented in the literature in the last few years, but none of them completely fits the requirements. Hsu *et al.* [19] presented two different designs both achieving 5 fA/√Hz (160 fArms at 1 kHz) but with different weaknesses: one has 560 kHz bandwidth but an insufficient gain of 100 MΩ; while the other has enough gain (4.7 GΩ) over a narrow bandwidth of 1 kHz. Moreover, both the circuits are realized using discrete components, so they are not the best solutions when highly-parallel (>1024 channels) HTS systems have to be designed. Jafari *et al.* [20], as well as Crescentini *et al.* [13], presented very low-noise CMOS frontends with high gain (>1 GΩ) and noise floor as low as 2 fA/√Hz (63 fArms at 1 kHz) and 3 fA/√Hz (95 fArms at 1 kHz) respectively, but they are limited in acquisition bandwidth, which was lower than 10 kHz. Rosenstein *et al.* [21] described a fast current readout IC for high-throughput DNA sequencing; the circuit has more than 1 MHz bandwidth but the noise floor is limited to 12 fA/√Hz (380 fArms at 1 kHz).

This paper presents a low-noise transimpedance amplifier realized in CMOS 0.35 μm technology with a measured input-referred noise as low as 4 fA/√Hz (133 fArms at 1 kHz), a gain of 2.25 GΩ and 100 kHz bandwidth. The transimpedance amplifier is based on integrator-differentiator scheme [14]. The CMOS implementation is scalable in terms of the number of concurrently acquired channels while minimizing the stray input capacitance and interference, with benefits in the noise performance since the noise is linked to the input capacitance [13,14]. An integrator-differentiator scheme provides a current sensing interface with the lowest noise floor, but suffers from saturation of the integrator stage [14]. To avoid saturation while maintaining a wide acquisition bandwidth and limiting the noise sources, we propose a periodic reset of the readout circuits at frequency *f_R_* with A/D sampling at frequency *f_S_ >> f_R_*, disregarding the reset behavior. In this way the folding noise due to sampling is reduced and the bandwidth is not limited by the reset. A second-order delta-sigma (ΔΣ) analog-to-digital converter (ADC) oversamples the signal at 10 MHz and generates a 1-bit 10 MS/s digital stream that is decimated by digital FIR filter implemented on a FPGA. This solution simplifies the signal routing when concurrently acquiring a great number of channels, and gives a flexible bandwidth-noise trade-off to the user by acting on the oversampling ratio (OSR) parameter in the decimator filter [22]. The system also integrates a digital offset cancellation loop (OCL) balancing any voltage offset from Ag/AgCl electrodes. The amplifier has been validated, together with microfluidic devices by measuring the activity of three different ion channels: gramicidin-A, α-haemolysin and KcsA potassium channels.

Section 2 briefly presents the overall platform and the microfluidic devices then describes the implementation of the CMOS transimpedance amplifier circuit with detailed noise analysis. Finally, Section 3 reports experimental measurements and validation of the proposed readout circuit.

## 2. Proposed Transimpedance Amplifier

### 2.1. Ion Channel Recording Platform

**Figure 2:**
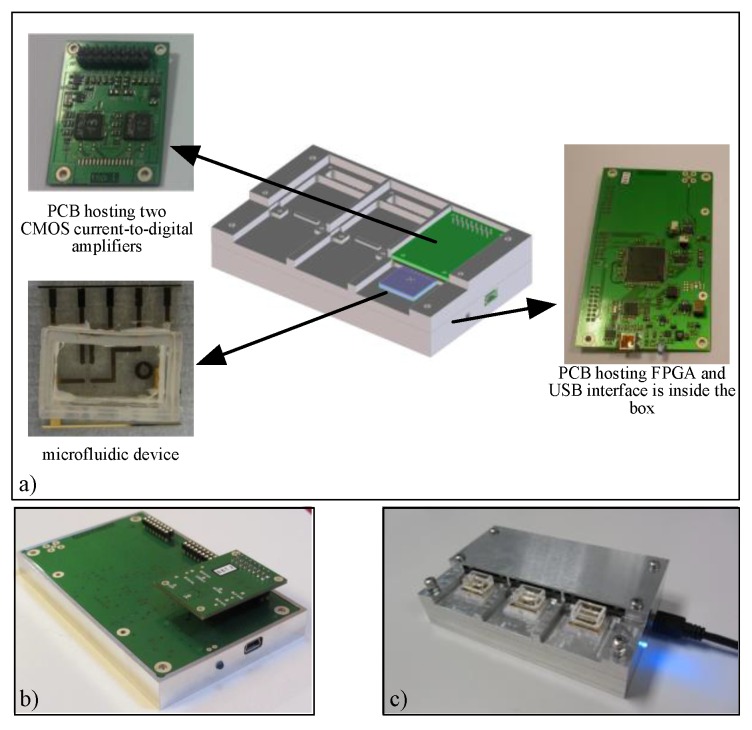
(**a**) Photograph of the 12-channel parallel recording platform highlighting each element: three small PCBs with two CMOS current-to-digital amplifiers described in this paper, three 4-channel microfluidic devices [12], and a PCB with FPGA and USB interface which is housed in the metal box; (**b**) Photograph of the platform showing board connections; (**c**) Photograph of the final platform with the metal box used for shielding.

The complete ion channel recording platform is able to concurrently acquire 12-channels, and is composed of (Figure 2):

- Three disposable microfluidic devices manufactured on a glass substrate holding 4 BLMs each [12].
- A small PCB hosting two CMOS 2-channel low-noise current-to-digital amplifiers that can measure pA currents.
- A motherboard with a digital control unit implemented in a Field Programmable Gate Array (FPGA) [11].

All the components are integrated onto a single platform, offering a fully scalable acquisition system. The system architecture was presented for the first time in [23], while the ability to concurrently acquire multiple channels was previously published in [11] and [13]. This paper focuses on the design rationale of the analog frontend of the CMOS current-to-digital amplifier. For a description of the parallel microfluidic platform refer to [11,12,13].

**Figure 3:**
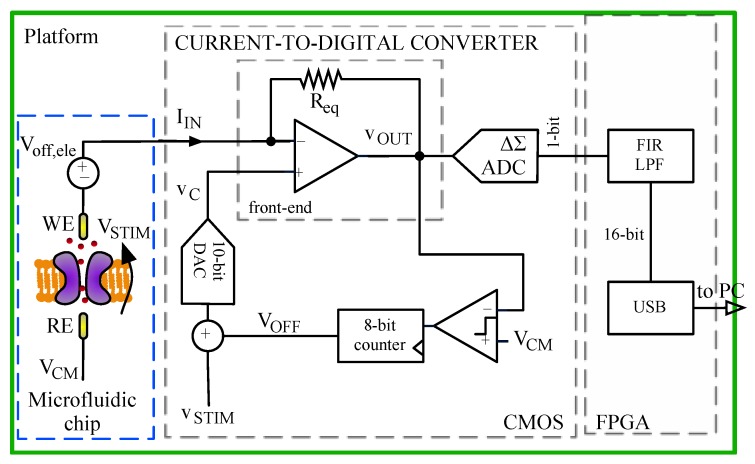
Block diagram of the system. A lipid bilayer is formed in a microfluidic chip, with integrated Ag/AgCl electrodes. Reference Electrode (RE) is tight to *V_CM_* while working electrode (WE) is connected to the input of the transimpedance amplifier. The CMOS transimpedance amplifier acquires the input current *i_IN_* and digitizes it into a 1-bit high-frequency delta-sigma modulated stream. It also compensates for electrode and opamp offset by means of a digital compensation loop that is activated at the beginning of every experiment. The ADC output is filtered and decimated by a digital FIR filter implemented on a FPGA. Data communication with PC is via a USB link. Virtual short circuit realized by the input LNA is used to apply a stimulus voltage *v_STIM_* to the BLM.

A block diagram of the platform is shown in Figure 3. The functionality of the system is as follows. The CMOS IC applies a voltage stimulus *V_STIM_* to the ion channel through the low-noise amplifier (LNA) virtual short circuit; this stimulus could be either a constant voltage or a time-varying voltage. The ionic current flowing through the ion channel is translated into an electronic current by Ag/AgCl electrodes in the microfluidic device. The CMOS IC acquires the input current *I_IN_* and digitizes it into a 1-bit data stream. It uses a novel scheme for the transimpedance amplifier and a 2nd order ΔΣ modulator targeting 16-bit resolution for the A/D conversion. The analog-to-digital converter (ADC) output is filtered and decimated by a FIR filter implemented on a FPGA. Finally, PC communication is via a USB link.

An internal digital loop compensates for any input voltage offset from the Ag/AgCl electrodes (*V_off,ele_* in Figure 3) [13]. This offset cancellation is done at the beginning of each experiment as follow:

- Read the front-end output voltage *V_OUT_*;
- Compare *V_OUT_* with the reference voltage *V_CM_*;
- Change DC voltage *V_OFF_* so that it becomes equal to *V_CM_* + *V_off,ele_*. (Note reference electrode is tight to *V_CM_*).

Final voltage *V_C_* applied to the LNA positive input is given by *V_C_* = *V_STIM_* + *V_OFF_* so that *V_OFF_* counteracts the electrode offset, while *V_STIM_* appears as the voltage drop across bilayer membrane. The system is fully programmable via SPI. Two input ranges are implemented (±20 nA and ±200 pA) with maximum acquisition bandwidths of 100 kHz.

### 2.2. Microfluidic Device

**Figure 4:**
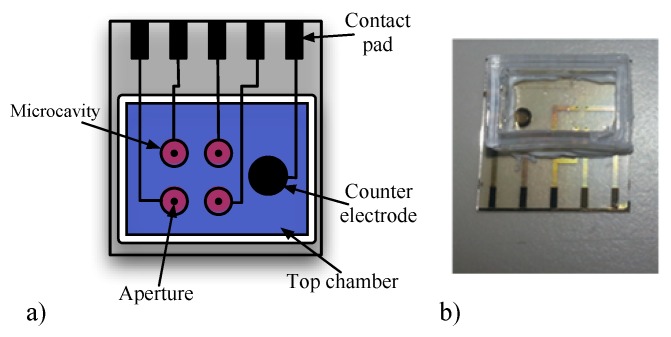
(**a**) Diagram showing the microfluidic device. The counter electrode sets the potential of the top fluid chamber, which is common to every BLM. There are four separate microcavities each with individual bilayers and separate integrated Ag/AgCl electrodes; (**b**) A photograph of the microfluidic device.

The microfluidic device holds up to four separate BLMs and is manufactured on a glass substrate [12]. It has dimensions of 15 × 15 mm with integrated Ag/AgCl electrodes. Bilayers were formed over apertures of approximately 100 μm diameter (Figure 4) [12,13]. The measured capacitance of a bilayer suspended across a 75 μm diameter aperture is typically 15–30 pF. Complete description of the microfluidic device can be found in [12].

### 2.3. Sensing Frontend Rationale

**Figure 5:**
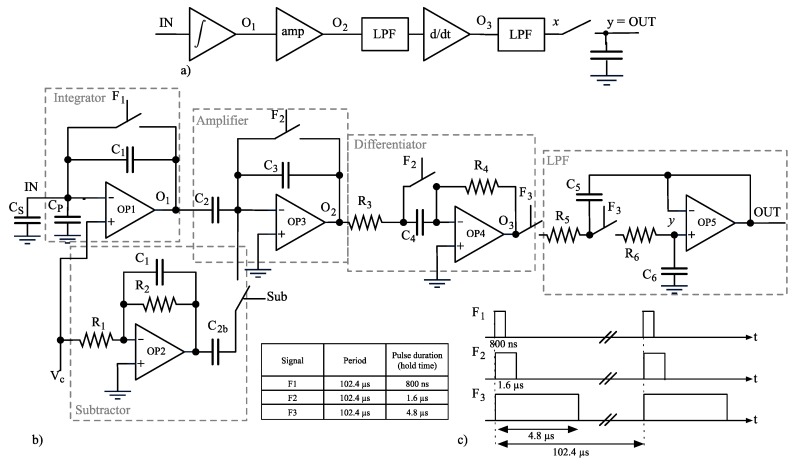
(**a**) Block scheme of the proposed frontend. The signal direct path is composed of integrator, voltage amplifier, differentiator and active LPF; (**b**) Full schematic diagram of the frontend, where a subtractor has been added to eliminate derivative component of stimulus signal from the final output. This subtractor stage can be activated/deactivated using the control signal Sub; (**c**) Timing behavior of signals F1, F2 and F3.

The front-end is based on integrator-differentiator scheme offering maximum noise performance due to the input integrator stage [14]. Figure 5 shows a schematic diagram of the complete front end. The direct signal path is composed of a current integrator, a capacitive voltage amplifier, a continuous-time (CT) differentiator outputting a voltage directly proportional to the input current *I_IN_*, and a Sallen-Key low-pass filter. Integrator, voltage amplifier and differentiator are periodically reset to avoid saturation, while the Sallen-Key filter holds the output voltage *v_OUT_* during reset.

**Figure 6:**
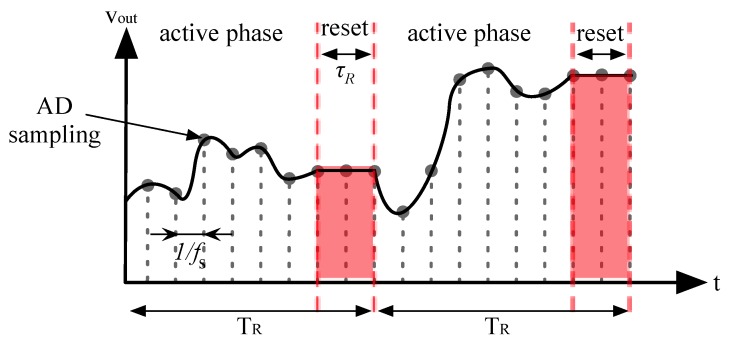
Timing behavior of the system. The frontend resets every period *T_R_* = 102.4 μs. During the reset, the output voltage is held at a constant value. AD sampling frequency is n-times higher than the reset frequency.

It is possible to discriminate two different phases as shown in Figure 6:

- Active phase. During this phase $v_{OUT}\left(t\right)=R_{eq}\cdot i_{IN}\left(t\right)$ where *R_eq_* is the equivalent trans-resistance of the amplifier, which is given by:
  (1) $$R_{eq}=\frac{C_{2}C_{4}}{C_{1}C_{3}}R_{4}$$
- Reset phase. During this phase the output voltage is kept constant while the rest of the circuit reset.

This 2-phase behavior is controlled by signal F3 internally generated from an external 80 MHz clock. To minimize the effect of charge injection, the control signals F1, F2 and F3 are designed to start at the same time but stopping one after the other, and the switches are realized by transmission-gates with dummy elements.

The proposed architecture differs from a standard discrete-time transimpedance amplifier, as defined in [14], since the sampling frequency *f_S_* is unrelated to the reset frequency *f_R_ = 1/T_R_*; specifically *f_S_* is greater than *f_R_*. In this way the acquisition bandwidth is not limited by the periodic reset but the noise becomes cyclostationary. Detailed analysis of the effects of cyclostationary properties of noise is discussed in Section 2.7.

Another important limitation on the maximum frequency is given by the bandwidth of the integrator that is almost equal to:

(2) $$BW_{Integrator}\sim GBW\frac{C_{1}}{C_{S}+C_{P}}$$

where *GBW* is the unity gain bandwidth of the OTA, *C_S_* is the capacitance of the microfluidic setup with the BLM, and *C_P_* is the parasitic capacitances due to interconnects and input stage of the transimpedance amplifier. The combination of Equations (1) and (2) sets a trade-off on the value of the feedback capacitance *C_1_* that should be small enough to maximize *R_eq_* and minimize input noise (see Section 2.7), but large enough to speed up the integrator. *C_1_* was set to 1 pF, where GBW = 92 MHz, *C_P_* is of the order of a few pF, and *C_S_* is expected to be in the range 40–80 pF [12]. With these parameters, a 1 MHz bandwidth of the integrator is obtained. This value is ten times higher than acquisition bandwidth of the entire system and ensures a fast settling of the integrator after reset. Note that now the acquisition bandwidth is not limited by periodical reset but only by bandwidth of the OTA and parasitic capacitances as reported in Equation (2). The reduction of trans-resistance *R_eq_* due to the chosen value for *C_1_* is compensated by the gain of the voltage amplifier stage placed between integrator and differentiator.

Timing characteristics of the reset phase, which are duration *τ_R_* and period *T_R_*, affect both the signal and noise. During the reset phase, the output voltage is disconnected from the input, hence the system does not see what the input current actually is, which leads to loss of information. As a result, *τ_R_* must be minimized while *T_R_* must be maximized. The same conclusion comes from noise analysis (see Section 2.7). Note that the reset period *T_R_* has an upper limit given by saturation of the first two OTAs. Assuming a maximum 200 pA DC input current (*∆I_IN_*) flowing through the input, then the system saturates after a time *T_SAT_* given by:

(3) $$T_{SAT}=C_{1}\frac{\Delta V_{O1}}{\Delta I_{IN}}$$

where *∆V_O1_* is the maximum allowed voltage swing at the integrator output, which is equal to:

(4) $$\Delta V_{O1}=\Delta V_{O2}\frac{C_{3}}{C_{2}}=\Delta V_{OUT}\frac{C_{3}}{C_{2}}$$

where we assumed *∆V_O2_ = ∆V_OUT_* since at low frequency the gain is set by the first two stages. Therefore the reset period *T* should be:

(5) $$T\leq T_{SAT}=\frac{C_{1}C_{3}}{C_{2}}\frac{\Delta V_{OUT}}{\Delta I_{IN}}=\frac{C_{1}C_{3}}{C_{2}}\frac{C_{2}C_{4}}{C_{1}C_{3}}R_{4}=C_{4}R_{4}$$

where we assumed a maximized full-scale, that is:

(6) $$\Delta V_{OUT}=R_{eq}\Delta I_{IN}$$

Equation (5) shows how the reset period *T_R_* is linked to the time constant *C_4_R_4_*; thus *C_4_* and *R_4_* must be maximized. Once *C_1_, C_4_* and *R_4_* are chosen, the ratio *C_2_/C_3_* is given by the combination of Equations (5) and (6). The resistor *R_3_*, and capacitor *C_4_*, creates a first-order low-pass filter, reducing the noise before differentiation.

A list of circuit parameters is reported in Table 1. The output voltage full-scale *∆V_OUT_* is fixed at ±450 mV by the OTA, thus *R_eq_* = 2.25 GΩ for *∆I_IN_* = ±200 pA and *R_eq_* = 22.5 MΩ for *∆I_IN_* = ±20 nA.

### 2.4. ADC

**Figure 7:**
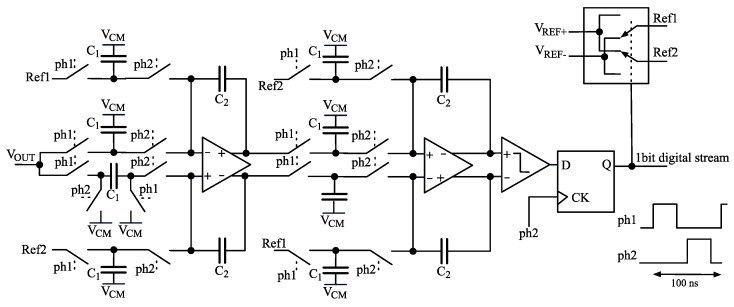
Schematic diagram of the second-order ΔΣ converter. V*_CM_* is half the power supply.

The ADC is integrated in the CMOS current-to-digital converter as shown in Figure 3. The scheme of the ADC is standard and it is reported in Figure 7; it is a switched-capacitors second-order ΔΣ converter operating between voltages V_REF+_ = 2.1 V and V_REF−_= 1.2 V. Timing signals, *ph1* and *ph2*, are two non-overlapping 10 MHz signals generated from an external 80 MHz clock. The use of a second-order ΔΣ converter allows keeping the quantization noise below the thermal noise of the input front-end thanks to ΔΣ noise shaping. The output of the ADC is a 10 MHz 1-bit data stream that is filtered and downsampled by a FIR digital filter implemented in the FPGA.

### 2.5. Stimulus Generation and Offset Compensation

The offset potential arising from the electrode-electrolyte interface can be of the order of tens to hundreds of millivolts, following the Nernst equation [24]. The dispersion of actual value of the offset potential around the nominal value strongly depends on type of the electrode and fabrication technique. Moreover, microfabricated electrodes suffer from higher instability and dispersion due to small sizes of the electrode and low control of process parameters [24,25,26].

**Figure 8:**
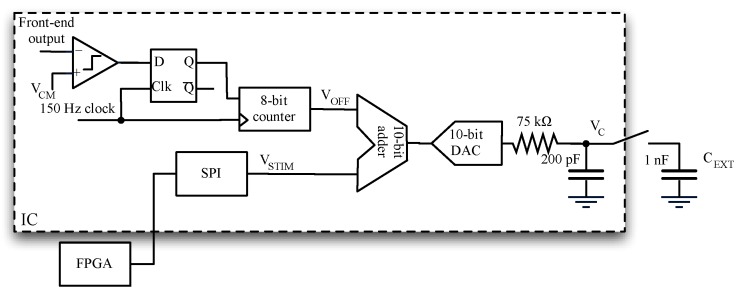
Architecture of the offset compensation loop. The offset compensation voltage *V_OFF_* is generated comparing the output of the frontend *v_OUT_* with *V_CM_* and then incrementing a 8-bit counter. Stimulus signal *v_STIM_* comes from the FPGA and is summed to *V_OFF_* and fed to a DAC so as to create voltage *v_C_* A passive LPF filter at DAC output limits the noise.

This offset potential generates offset current that limits the acquisition range or even causes saturation of the amplifier, since the equivalent transresistance is very high. For instance; 200 mV offset over 1 GΩ channel resistance leads to 200 pA current that saturates the transimpedance amplifier when working in the 200 pA range. To cope with this, the OCL is activated at the beginning of each experiment [27]. It compares *V_OUT_* with the reference voltage *V_CM_*, which is the bias voltage of the reference electrode, and generates a DC voltage *V_OFF_* that is applied to the positive input of the integrator:

(7) $$V_{OFF}=\frac{V_{CM}\left(1+\frac{R_{eq}}{R_{S}}\right)+\frac{R_{eq}}{R_{S}}V_{off,ele}}{\left(1+\frac{R_{eq}}{R_{S}}\right)}\approx V_{CM}+V_{off,ele}$$

where *R_S_* is the equivalent resistance of the ion channel. Under the assumption *R_eq_* >> *R_S_* then the offset current is almost nulled, while a small offset current still remains in case of higher value of *R_S_*. Note that offset of the LNA is compensated along with offset of the electrodes. The compensation loop is realized by means of a comparator, a latch and an 8-bit up/down counter working at 150 Hz (Figure 8).

A time-varying stimulus signal *v_STIM_* is needed when working with voltage gated ion channels or for full characterization of ion channels. The signal *v_STIM_* is digitally generated by the FPGA and then sent to the CMOS amplifier through SPI interface, as shown in Figure 8. Both offset compensation and stimulus generation are addressed in the digital domain. Voltages *v_STIM_* and *V_OFF_* are added together and converted in analog domain by a 10-bit DAC to create the voltage *v_C_* that is applied to the positive input of the integrator (Figure 3):

(8) $$v_{C}=v_{STIM}+V_{OFF}$$

The 10 bits of the DAC must accommodate the swing for both *v_STIM_* and *V_OFF_*, limiting them to ±384 mV and ±128 mV, respectively. At the DAC output, a passive LPF filters out the high frequency noise. Extremely low noise acquisitions require an external capacitor of at least 1 nF. Note that a larger external capacitance reduces the noise as well as the bandwidth of the stimulus voltage. For instance, setting *C_EXT_* = 1 nF limits the bandwidth of *v_STIM_* to a few kHz.

### 2.6. Subtractor

The voltage *v_C_ = v_STIM_* + *V_OFF_* is applied to the DUT by means of the virtual short circuit imposed by the negative feedback of the integrator. Hence the integrator behaves like a non-inverting amplifier from the *v_C_* standpoint, and the voltage *v_01_* can be written as:

(9) $$v_{01}=\int \left(\frac{C_{S}+C_{P}}{C_{1}}+1\right)\frac{dv_{C}}{dt}dt=\int \frac{i_{IN}}{C_{1}}dt+v_{C}$$

Equation (9) states that *v_C_* signal directly propagates through the first stage as an unwanted additive component to the measured input signal. To avoid this effect, the subtractor stage multiplies *v_C_* by −1 and adds its output to integrator output (Figure 5). Obviously this stage adds noise but it is not needed for electrophysiology experiments requiring constant *v_C_*; hence, it is possible to activate or deactivate it using control signal Sub.

### 2.7. Noise Analysis

Noise models presented in [14] are not directly applicable to the proposed architecture since it is based on a combination of both continuous time (CT) and discrete time (DT) approaches. Hence a new model is derived, based on theory and methods described in [28,29]. The analog frontend can be simplified as shown in Figure 5a, where the following assumptions were made:

- all the stages prior to the sampling are treated as linear time-invariant systems;
- node *x* takes into account low-pass filtering done by the Sallen-Key but not the sampling; there is not a direct correspondence of node *x* in the schematic diagram (Figure 5b).
- node *OUT* is renamed into *y* to get more compact equations.

**Figure 9:**
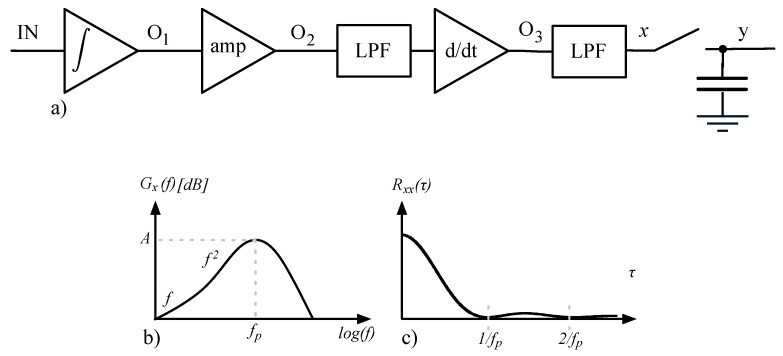
(**a**) Simplified block scheme of the proposed architecture. Noise analysis is based on this system simplification where sampling is decoupled from the Sallen-Key filter; (**b**) Qualitative sketch of the noise PSD at node *x*; (**c**) Qualitative sketch of noise autocorrelation function at node *x*.

The noise power spectrum density (PSD) at node *x* can be written as:

(10) Gx(f)~(2πf)2(C1+CIN)2Req2⋅en2¯⋅1(1+ffp)2

where *C_IN_ = C_S_ + C_P_*, *e_n_* is the input-referred noise source of the OTA, *f_p_* is the dominant pole of the system that is set by the LPF, and noises generated by voltage amplifier and differentiator have been neglected. Note that *G_x_(f)* has not a white shape but it rises with *f* where Flicker dominates, and with *f^2^* where thermal noise dominates (Figure 9a). Simplifying *G_x_(f)* to a triangular shape the autocorrelation function of *x(t)* becomes:

(11) $$R_{xx}\left(\tau \right)\sim Af_{p}{\text{sinc}}^{2}\left(f_{p}\tau \right)$$

where *A* is the peak value of *G_x_(f)*. The voltage at node *y* can be seen as $v_{y}\left(t\right)=y^{\prime }\left(t\right)+y^{\prime \prime }\left(t\right)$:

(12) $$\begin{array}{l} y^{\prime }\left(t\right)=\left\{ \begin{array}{ll} v_{x}\left(t\right) & \text{ in active phase}\\ 0 & \text{ in reset phase} \end{array}\right.\Rightarrow {\text{ y}}^{\prime }=v_{x}\left(t\right)\sum_{n}p\left(t-nT\right)\;\\ y^{\prime \prime }\left(t\right)=\left\{ \begin{array}{ll} 0 & \text{in active phase}\\ v_{x}\left(nT\right) & \text{in reset phase} \end{array}\right.\;\Rightarrow {\text{ y}}^{\prime \prime }\left(t\right)=\sum_{n}v_{x}\left(nT-T_{R}\right)\cdot \left[1-p\left(t-nT\right)\right] \end{array}$$

where *p(t)* is a step function equal to zero in the reset phase and equal to 1 for every other time. Signal *y′(t)* takes into account the linear CT behavior of the system, although periodicity creates cyclostationary properties [30], while *y″(t)* takes into care the DT sampling behavior. The noise PSD at node *y* can be written as [31]:

(13) Gy(f)=Gy′(f)+Gy″(f)+2Re{F[〈Ry′y″(t,τ)〉TR]}

where *F* denotes the Fourier transform, *R_y′y″_* is the cross-correlation function and *T_R_* is the reset period.

The first term in Equation (13) can be expanded as:

(14) $$G_{y^{\prime }}\left(f\right)=\frac{1}{T_{R}^{2}}\sum_{n}{\left(T_{R}-\tau_{R}\right)}^{2}{\text{sinc}}^{2}\left[\frac{n\left(T_{R}-\tau_{R}\right)}{T_{R}}\right]G_{x}\left(f-\frac{n}{T_{R}}\right)$$

where *τ_R_* is the pulse duration of F3. Note that periodicity of *y′* leads to folding of the noise PSD. However the sinc function is around zero for all *n* ≠ *0* since *τ_R_ << T_R_;* hence Equation (14) can be simplified to:

(15) $$G_{y^{\prime }}\left(f\right)=\frac{{\left(T_{R}-\tau_{R}\right)}^{2}}{T_{R}^{2}}G_{x}\left(f\right)≅G_{x}\left(f\right)$$

This simplification is even more valid when *τ_R_* tends to zero (*i.e.*, pure CT behavior) while the whole term in Equation (14) goes to zero when *τ_R_* tends to *T_R_* (*i.e.*, pure DT behavior).

The second term in Equation (13) is the noise PSD of a standard sampling process, hence it can be written as [14,31,32]:

(16) $$G_{y^{\prime \prime }}\left(f\right)=\frac{\tau_{R}^{2}}{T_{R}^{2}}{\text{sinc}}^{2}\left(f\tau_{R}\right)\cdot \sum_{k}G_{x}\left(f-\frac{k}{T_{R}}\right)$$

The third term in Equation (13) needs a little more derivation. Denoting random variables with upper case letters, then the cross-correlation function can be written as:

(17) $$\begin{array}{l} R_{y\prime y\prime \prime }\left(t,\tau \right)=E\left[Y\prime \left(t\right)Y\prime \prime \left(t\right)\right]\\ =E\left[X\left(t\right)\sum_{n}p\left(t-nT_{R}\right)\sum_{m}X\left(mT_{R}-\tau_{R}\right)\left[1-p\left(t-\tau -mT_{R}\right)\right]\right]\\ =R_{xx}\left(\tau_{R}\right)\sum_{n}\sum_{m}\delta \left(t-mT_{R}\right)p\left(t-nT_{R}\right)\left[1-p\left(t-\tau -mT_{R}\right)\right] \end{array}$$

Since *R_xx_(τ)* has the form expressed in Equation (11), then *R_xx_(τ_R_)* is around zero for *τ_R_ > 1/f_p_*. In our case *τ_R_ = 4.8* μs and *f_p_* is around 710 kHz, hence we neglect the cross-correlation term in Equation (13). If *1/f_p_ > τ_R_* this simplification is not valid any longer and Equation (17) should be considered.

Using all the above, we simplify Equation (13) to:

(18) $$G_{y}\left(f\right)=G_{x}\left(f\right)+{\left(\frac{\tau_{R}}{T_{R}}\right)}^{2}{\text{sinc}}^{2}\left(f\tau_{R}\right)\sum_{k}G_{x}\left(f-\frac{k}{T_{R}}\right)$$

Note that high frequency thermal noise gives the main contribution to folding noise while Flicker noise can be easily ignored in both the terms because of its frequency shape (see Figure 9b). The first term in Equation (18) is the noise PSD before sampling, while the second term in Equation (18) is the folding of high frequency noise due to the reset process. Unfortunately the folding term cannot be easily simplified using the undersampling ratio (USR) as done in [14,28], because *G_x_(f)* has not a standard white shape. Finally the input-referred noise is given by:

(19) $$\bar{i_{in}^{2}}\left(f\right)=\frac{G_{y}\left(f\right)}{R_{eq}^{2}}≅\frac{G_{x}\left(f\right)+{\left(\frac{\tau_{R}}{T_{R}}\right)}^{2}{\text{sinc}}^{2}\left(f\tau_{R}\right)\sum_{k=0}^{USR}G_{x}\left(f-\frac{k}{T_{R}}\right)}{R_{eq}^{2}}$$

Equation (19) was implemented in Matlab by solving the summation for *k* up to *USR* = *πf_P_T_R_*. Matlab analysis reveals that folding noise dominates the first term in Equation (19), although it is multiplied by a pre-factor (*τ_R_/T_R_)^2^* that is much less than 1. Note that this pre-factor is near 1 in pure-DT approaches.

**Figure 10:**
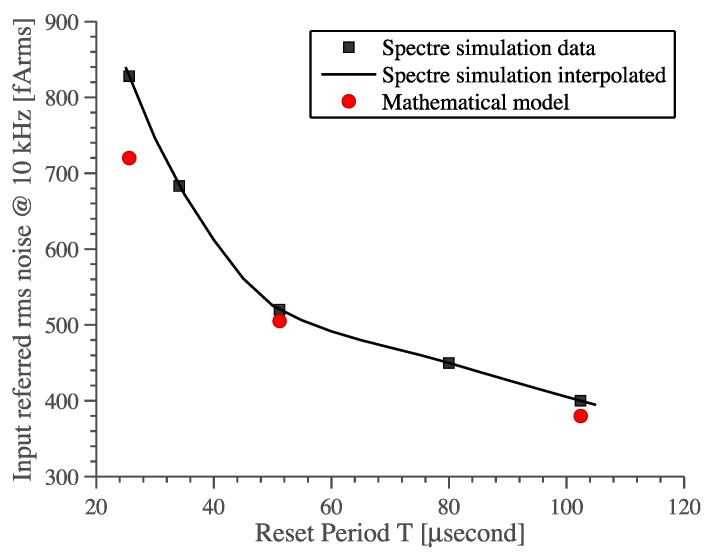
Effect the reset period T on the input-referred r.m.s. noise. Comparison of the proposed mathematical model with SpectreRF^®^ pss-noise analysis.

Equation (19) indicates some design considerations:

- The most direct method of reducing folding noise is lowering the USR, which defines how many times the noise folds back into the baseband. This can be easily done by lowering *f_p_*, but this directly affects the bandwidth of the system and the sampling error [14]. Moreover, if *1/f_p_* becomes greater than the reset pulse duration *τ_R_* then Equations (18) and (19) are no longer valid, since cross-correlation power computed from Equation (17) must be taken into consideration.
- Another important parameter is the period *T_R_*, which appears in both USR and pre-factor. It should be small to lower USR while it should be big to lower the pre-factor (*τ_R_/T_R_)^2^.* Since *T_R_* is squared in the pre-factor term then it is better to make it as high as possible. This relation between noise and parameter *T_R_* is confirmed by periodic-steady-state noise (pss-noise) analysis and it is predicted by our mathematical model, see Figure 10.
- Another way of reducing the folding noise is to keep *G_x_(f)* as low as possible. This directly translates into using a low-noise OTA and lowering the input capacitance *C_IN_* as well as the feedback capacitance *C_1_*, creating a noise-bandwidth trade-off.

## 3. Experimental Results

### 3.1. Implementation

**Figure 11:**
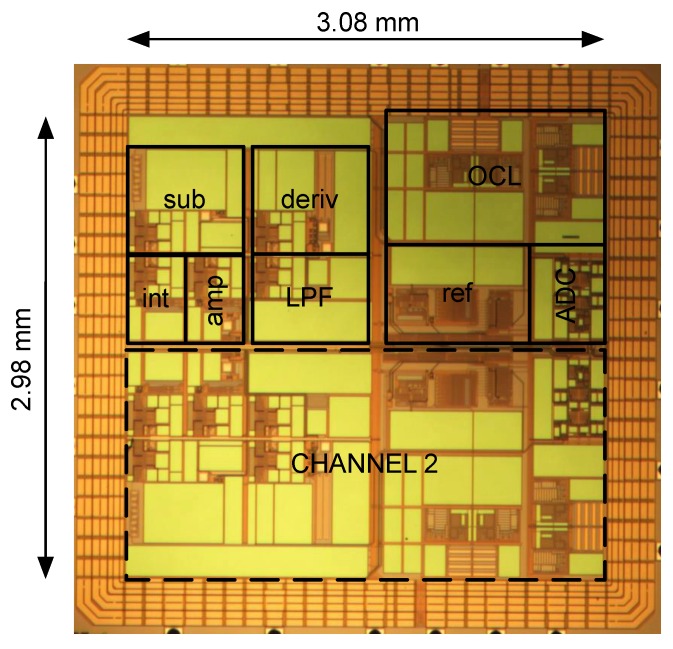
Chip microphotograph.

The system has been implemented in 0.35 μm CMOS technology. A microphotograph of the ASIC is shown in Figure 11. It has two separate current frontends occupying a total area of 9.2 mm^2^. The two-core design was dictated by the need to implement a prototype compact parallel recording platform, with 12 channels acquired concurrently [13]. A single core consumes a total of 41 mW. This power consumption includes all the circuits implemented in the CMOS chip, *i.e.*, analog frontend, ADC, DAC, voltage references, clock buffering and digital circuits. The system can acquire signals up to 100 kHz at the highest resolution.

### 3.2. Noise Measurements

The −3 dB bandwidth reported in following figures is set by the digital FIR filters at the output of the ΔΣ ADC. We implemented 150 taps FIR filters with triangular windowing at different cut-off (−6 dB) frequencies (10 kHz and 200 kHz). The −3 dB bandwidth of these filters is 7.5 kHz and 175 kHz respectively. Note that the acquisition bandwidth can be theoretically increased up to half the ADC sampling frequency (*i.e.*, 5 MHz), losing in resolution. However, the Sallen-Key LPF in Figure 5 and the quantization noise practically limit the maximum bandwidth to 710 kHz and 200 kHz, respectively. For bandwidths higher than 200 kHz, *i.e.*, OSR lower than 26, the quantization noise dominates over the thermal noise reported hereafter.

**Figure 12:**
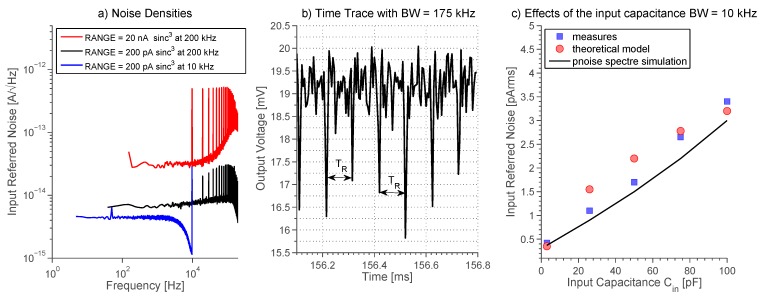
(**a**) Open-input input-referred noise measured at two different ranges (20 nA and 200 pA). The noise is as low as 4 fA/√Hz in the best condition (*i.e.*, range = 200 pA and digital filtering at 10 kHz with a sinc^3^ LPF), while raises to 6 fA/√Hz at 200 pA range and digital filtering at 200 kHz. The highest noise floor, that is 40 fA/√Hz, is shown in the wider 20 nA range. Digital FIR filters with triangular windowing and 150 taps were used; (**b**) Short-time trace recorded at bandwidth = 175 kHz showing periodic spikes every *T_R_* = 102.4 μs. This spikes are linked to the period reset activity; (**c**) Relation between input-referred r.m.s. noise at 10 kHz and input capacitance *C_in_*, which takes into care microfluidic device together with BLM. Measured values are compared with simulations and theoretical estimation, validating Equation (19).

The input-referred noise PSD is computed by dividing the output noise PSD by the square of the equivalent transresistance *R_eq_*. Figure 12a shows the open-input input-referred noise measured at both the 200 pA and 20 nA input ranges with deactivated subtractor stage. The system has an input noise as low as 4 fA/√Hz in the 7.5 kHz bandwidth, and 6 fA/√Hz in the 175 kHz bandwidth, proving the low-noise capability and the wide acquisition bandwidth. Noise PSDs are flat at low frequencies since folding noise dominates, as discussed in Section 2.7, while they rise at high frequencies where the CT term dominates, as usual in transimpedance amplifiers [14].

Table 2 compares the noise model given by Equation (19) with measured and simulated r.m.s. noise over a 10 kHz bandwidth. The theoretical value was obtained by implementing Equation (19) in Matlab and solving the summation for *k* up to USR = *πf_P_T*. The parameters are the followings: *f_p_* = 710 kHz, *T_R_* = 102.4 μs, *τ_R_* = 4.8 μs, *C_1_* = 1 pF, *R_eq_* = 2.25 GΩ, *e_n_* = 3 nV/√Hz. Noise simulation was done using SpectreRF^®^, which takes into account noise folding and cyclostationary properties of the system. Note that *C_IN_ = C_P_ = 3* pF was used in both mathematical model and simulations to count for stray capacitances facing to the input node, such as capacitive effects due to pad, bonding wires, pin, *etc.* This value was indirectly estimated from measurements and parasitic extraction.

Figure 12a also shows the input-referred noise current recorded at maximum bandwidth with the 20 nA input range selected. The noise floor is ten times higher than noise measured at the 200 pA range. Spikes around 10 kHz and multiple frequencies results from the periodicity of the system due to the cyclostationary nature of the output noise [30]. This behavior can be seen as periodic spikes in the time-domain (Figure 12b). The noise is closely dependent on *C_IN_* as seen in Equation (10) and demonstrated by measurements shown in Figure 12c. This result confirms the need for small microfluidic device and integrated electronic readout placed as close as possible to the microfluidic chip. All the noise PSDs described above refer to measurements done in the optimum condition of deactivated OCL and constant voltage *v_C_*.

### 3.3. Offset Compensation Loop and Subtractor

**Figure 13:**
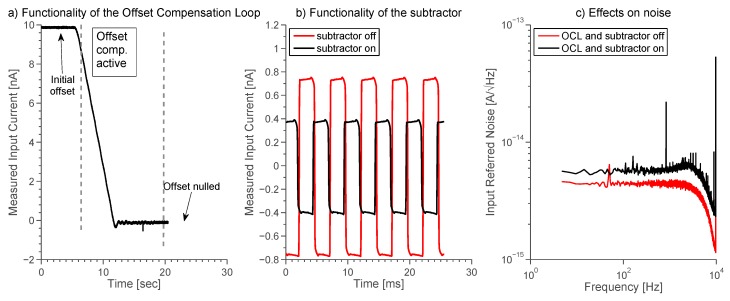
(**a**) Functionality of the OCL. An offset in the nA range usually exists at the beginning of the experiment. The OCL changes the DC component of the applied stimulus *v_C_* until a zero current is recorded (*i.e.*, *V_OUT_* = *V_CM_*); (**b**) Functionality of the subtractor. A 10 pF capacitor is connected to the input while a 100 mVpp triangular wave at 200 Hz is applied as *v_C_* signal leading to a 800 pApp current square wave flowing through the input. The input current is overestimated when the subtractor is turned-off; (**c**) Effect of the OCL on the input-referred noise. When the OCL is activated and a stimulus signal *v_STIM_* is applied, the noise rises from 4 fA/√Hz to 6 fA/√Hz due to noise sources in the OCL. Note that *v_C_* is filtered with an external 10 nF capacitance.

Demonstration of the effectiveness of the OCL is reported in Figure 13a. The presence of the electrode offset causes a nA current to flow into the transimpedance amplifier. The OCL changes the DC component of the stimulus voltage so as to counteract the electrode offset and apply a zero DC voltage to the BLM.

Subtractor stage has been tested connecting 10 pF to the input node and applying a 100 mVpp triangular wave as *v_C_* signal. Under this condition, a 400 pA (800 pApp) square wave current flows through the input node. The amplifier works at ±20 nA range and 7.5 kHz bandwidth. Figure 13b reports the estimated input current, computed referring the output of the FIR filter back to the input by means of the equivalent transresistance, when subtractor stage is either deactivated or activated. Following the analysis described in section II, the estimated input current is given by:

(20) $${\hat{i}}_{IN}=\frac{v_{out}}{R_{eq}^{}}=\left(1+\frac{C_{1}}{C_{S}}\right)i_{IN}$$

In the former case, the input current is overestimated by a factor of 2, while the latter case reports the correct 400 pA value, proving the functionality of the subtractor stage. The activation of the OCL and the subtractor adds noise sources in the circuit increasing the input-referred noise, as shown in Figure 13c.

### 3.4. Ion Channel Recording

The system was tested by acquiring data of single ion-channel currents. The measurement setup consists of a single microfluidic device with its own ASIC frontend mounted on the platform shown in Figure 2. Validation tests were done with three different kinds of ion-channel: gramicidin-A, α-haemolysin and KcsA potassium channel.

#### 3.4.1. Gramicidin-A

**Figure 14:**
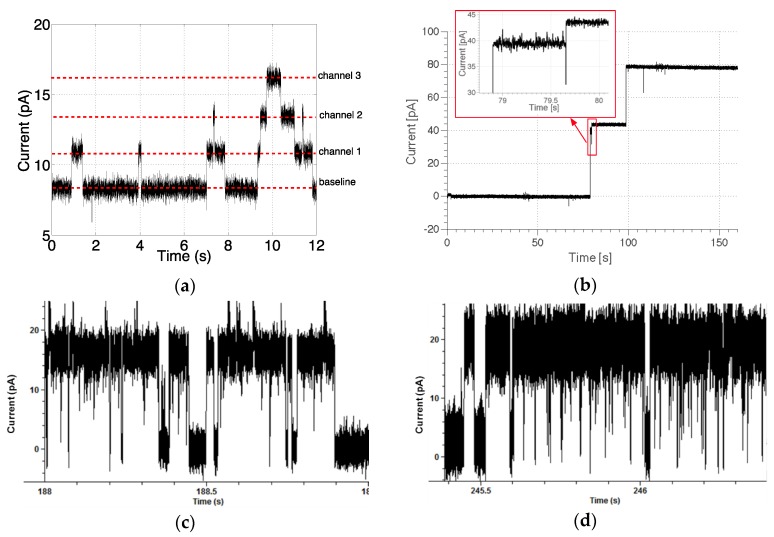
(**a**) Data from gramicidin-A channels. Applied potential = 100 mV and the acquisition has been performed at 625 Hz; (**b**) Current trace showing the insertion of two α-HL nanopores. Experiments performed with 2.5 μg/ml of α-HL protein in 1 M of KCl solution. Acquisition performed at 200 pA range with post-processing filtering at 1.25 kHz; (**c**) KcsA recording at 125 mV with data filtered at 5 kHz and (**d**) 10 kHz. The buffer was 150 mM KCl, pH 4.0.

The microfluidic device was filled with buffer solution (1 M KCl, 10 mM HEPES, pH 7.4) and bilayers formed by painting a 10 mg/mL solution of phospholipid (1,2-diphytanoyl-*sn*-glycero-3-phosphocholine, DPhPC, Avanti Polar Lipids, Alabaster, AL, USA) in decane over the apertures. Figure 14a shows current traces for gramicidin-A ion-channels with an applied potential of 100 mV. Data was acquired at 625 Hz, and shows the opening of three independent ion-channels (each current step corresponds to a single gramicidin A dimer). The typical ion channel current step is about 2.6 pA, which corresponds to a conductance of ~26 pS and matches literature values for gramicidin-A [33,34]. This simple test proves the ability of the system to acquire low current signals involved in standard ion-channel monitoring.

#### 3.4.2. α-Haemolysin

To demonstrate the capability of the system to record larger current steps, a 2.5 μg/mL solution of the protein α-HL was added to the top aqueous compartment. Figure 14b shows current steps of the order of 50 pA, indicative of the insertion of single α-HL nanopores, at an applied potential of 50 mV. A small number of current steps is to be expected because α-HL does not exhibit a transition between a closed and an open state. Again, the magnitude of the current steps is in reasonable agreement with literature values for α-HL in 1 M KCl solution [35,36].

#### 3.4.3. KcsA Potassium Channel

To demonstrate fast gating characteristic, the wild type KcsA channel, which has rapid closing and opening events was used. The BLM was made from a mix of POPC and POPG (1:1) lipids, due to the influence of PG on channel gating [16]. KcsA activates only in acidic pH, therefore both the top and bottom compartment was filled with 150 mM KCl, 10 mM HEMS, pH 4.0. A 5 μL suspension of proteoliposomes containing KcsA channels (1000:1 lipid to protein ratio) was added to the top compartments and the potential applied to the bottom compartment. Characteristically, KcsA displays long-lived ‘quiet’ or closed intervals and exhibits low open probabilities [16]. Single channel traces in symmetrical 150 mM K+ solutions, at 120 mV are shown in Figure 14c,d, where the current steps are consistent with previous published results [35]. The data shows the platform can record fast gating channels.

It was not possible to perform ion channel recording at higher bandwidths due to limitations in the microfluidic-biological setup; that are mainly capacitive loading and biological noise. In fact, the channel activity is barely visible even in the 10 kHz-filtered (−6 dB) acquisition of Figure 14d. Increasing the acquisition bandwidth to 100 kHz will lead to a three-fold higher noise, at least.

### 3.5. State-of-the-Art Comparison

**Figure 15:**
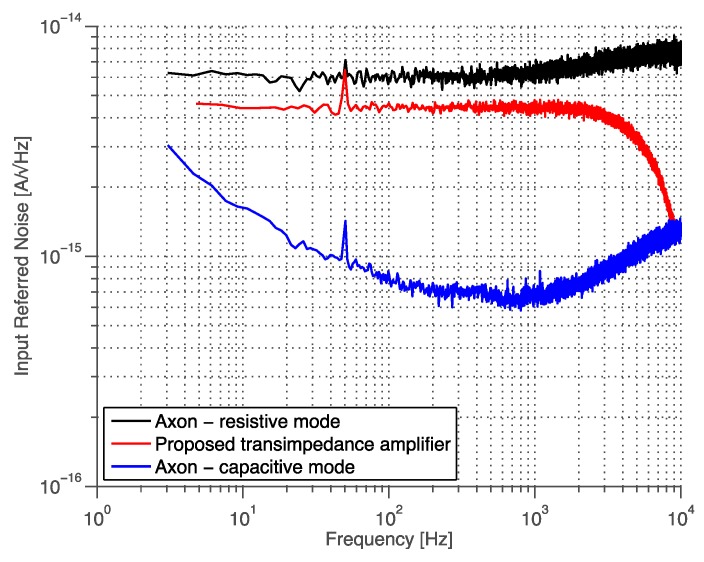
Input-referred noise compared with benchmark instrument for biological current acquisition. The peaks shown in all the lines are due to spurious coupling from the 50 Hz powerline.

The benchmark instrument for low-noise acquisition of small currents (<1 nA) is the Axon Axopatch 200B (Molecular Devices, Sunnyvale, CA, USA), which has an input-referred noise of 6 fA/√Hz in resistive mode, and 0.7 fA/√Hz in capacitive mode [18]. A comparison between Axopatch and the transimpedance ASIC is shown in Figure 15. Our system has comparable performances both in term of bandwidth and noise but integrated in a single silicon chip.

Comparison with state-of-the-art low-noise CMOS current interfaces is reported in Table 3. Our system has a state-of-the-art noise performance (6 fA/√Hz) together with wide acquisition bandwidth (100 kHz) and high gain (2.25 GΩ), meeting all the requirements of BLM ion-channel recording. It also embeds the ADC simplifying the design of parallel acquisition platform. Note that power consumption is not a main concern for this type of application, providing it is lower than a few Watts. Ferrari *et al.* [37] achieves optimum noise performance and wide bandwidth but the output is still a current, needing a I/V stage that may introduce extra noise. Moreover, the system is shot-noise limited, showing a noise floor that increases with the input current. Reference [38] is the only CMOS transimpedance amplifier presenting low-noise (<10 fA/√Hz) and wide bandwidth (>100 kHz), but does not embed the ADC.

## 4. Conclusions

This paper presented a CMOS transimpedance amplifier based on the integrator-differentiator scheme for BLM ion-channel recording. The application requires low-noise current acquisition (~fA/√Hz), high transimpedance value (>1 GΩ) and wide acquisition bandwidth (>10 kHz) in a compact design that can be easily parallelized for a future implementation in HTS instruments. CMOS integration of the architecture is a key feature since parasitic capacitances on the input node strongly increase the electronic noise.

The integrator-differentiator scheme is periodically reset to avoid saturation but the output voltage is sampled at frequency *f_S_* > *f_R_* disregarding the reset behavior. In this way the circuit achieves wide acquisition bandwidth and low noise performance even at low frequencies. The final result is a current-to-digital converter meeting all the applications requirements: (i) noise floor less than 10 fA/√Hz (*i.e.*, 400 fA_rms_ at 10 kHz and 1.9 pA_rms_ at 100 kHz, both measured at ±200 pA range); (ii) 100 kHz bandwidth; (iii) transimpedance of 2.25 GΩ; (iv) power consumption of 41 mW per channel including the ADC. The proposed architecture is one of the faster transimpedance amplifiers in the literature and it offers state-of-the-art noise performance.

A full design rationale, together with noise analysis, was presented describing all the trade-offs and design options. The system has two acquisition ranges (±200 pA and ±20 nA), while the acquisition bandwidths can be easily changed setting the OSR of the digital FIR filters. The front-end can apply a stimulus voltage to the BLM through virtual short-circuit of the first OTA. This feature is very useful when working with voltage-gated ion channels. Moreover, an integrated digital offset compensation loop balances any offsets in the Ag/AgCl electrodes.

The current-to-digital converter has been embedded in a multi-channel heterogeneous platform, along with microfluidic chips for current acquisitions from ion channels. Gramicidin-A, α-haemolysin and KcsA potassium channels have been used to prove both the platform and the current-to-digital converter.

## Figures and Tables

**Table 1:** Values of system parameters.

Component	C_1_	C_2_	C_3_	C_4_	R_4_	R_3_	T_R_	*τ_R_*
Value	1 pF	22 pF	1 pF	102.4 pF	1 MΩ	2.2 kΩ	102.4 μs	4.8 μs

**Table 2:** Comparison of noise theory with simulation and measures.

	Theory (18)	Simulation	Measure
RMS NOISE at 10 kHz	380 fA	400 fA	420 fA
Conditions	C_IN_ = C_P_ = 3 pF	C_IN_ = C_P_ = 3 pF	Open-input

**Table 3:** State-of-the-art comparison.

*Paper*	*Noise floor @ Room Temperature*	*Embedded ADC*	*Analog Power Consumption*	*Digital Power Consumption*	*Input Capacitance for Characterization of Noise Floor*	*Operating Bandwidth [kHz]*	*Gain [GΩ]*	*Technology Node*
[21]	12 fA/√Hz	NO	-	-	-	10.000	<1	CMOS 0.13 μm
[20]	2 fA/√Hz	NO	3 μW	-	-	6	>1	CMOS 0.13 μm
[37]	0.5 fA/√Hz	NO	-	-	1 pF	1000	*^1^	-
[39]	6 fA/√Hz	NO	1.5 mW	-	7 pF	50	-	CMOS 0.5 μm
[38]	4 fA/√Hz	NO	45 mW	-	800 fF	10,000	0.06	CMOS 0.35 μm
[40]	11.6 fA/√Hz	NO	5.22 mW	-	-	1400	0.01	CMOS 0.18 μm
[13]	3 fA/√Hz@B = 625 Hz 12 fA/√Hz @B = 10 kHz	YES	20 mW	20 mW	3 pF	10	2.25	CMOS 0.35 μm
This work	4 fA/√Hz*^2^ @B = 7.5 kHz 6 fA/√Hz *^2^ @B = 175 kHz	YES	21 mW	20 mW	3 pF	100	2.25	CMOS 0.35 μm

*^1^ The output of this amplifier is a current; *^2^ Noise floor measured at ±200 pA range.
